# Brain Amyloid in Sporadic Young Onset Alzheimer’s Disease

**DOI:** 10.3233/ADR-220110

**Published:** 2023-04-06

**Authors:** Peter K. Panegyres, Peter Robins

**Affiliations:** a Neurodegenerative Disorders Research Pty Ltd, West Perth, WA, Australia; b School of Medicine, The University of Western Australia, Nedlands, WA, Australia; c Department of Nuclear Medicine and WA PET Service, Nedlands, WA, Australia

**Keywords:** Alzheimer’s disease, brain amyloid, sporadic, young onset dementia

## Abstract

**Background:**

Controversy exists as to the role of the amyloid-β (Aβ) peptide in the pathophysiology of Alzheimer’s disease (AD).

**Objective:**

To clarify the effect of age on Aβ deposition in sporadic AD by exploring the degree of amyloid burden in patients with sporadic young onset AD (YOAD).

**Methods:**

Patients were diagnosed with YOAD with dementia starting before the age of 65 years (N = 42; males = 20, females = 22). A cross-sectional analysis of amyloid binding using positron emission tomography (PET) imaging was performed using the C-Pittsburgh Compound B (PiB). The global standardized uptake value ratios (gSUVR) were examined using the Wilcoxon two-sample test, as were the cognitive scores between disease and healthy control populations. Differences in PiB retention in different anatomical areas were compared using the Kruskal-Wallis test. The contrast in *APOE* genotyping between groups was calculated with Fisher’s Exact Test.

**Results:**

Women had a median gSUVR = 2.68±0.73 and 73% had at least one *APOE* ɛ4 allele. Men had gSUVR = 2.37±0.54, with 80% having at least one *APOE* ɛ4 allele. The gSUVRs were significantly higher than the control populations for men and women and had significantly greater frequency of *APOE* ɛ4. Men and women analyzed together had significantly greater amyloid burden and *APOE* ɛ4 allele frequencies than controls, but no differences existed between them in gSUVR nor in the anatomical distribution of amyloid uptake.

**Conclusion:**

Men and women with YOAD have greater amyloid uptake than controls and have more *APOE* ɛ4 alleles. Our findings suggest that the Aβ peptide is operational in young onset dementia and driven by the *APOE* ɛ4 allele.

## INTRODUCTION

The amyloid-β (Aβ) peptide that forms the neuritic plaque of Alzheimer’s disease (AD) is a derivative of the amyloid-β protein precursor by the action of secretases, including presenilin [1]. It, along with the microtubular associated protein tau—which, in its hyperphosphorylated form, causes neurofibrillary tangles—form the pathological hallmarks of AD, along with neuronal loss. The mechanisms by which these proteins accumulate in the brain of patients with sporadic AD are unknown. Rare genetic mutations leading to familial genetic AD, like the presenilin mutations which are a component of the *γ*-secretase complex, result in the aggregations of Aβ and secondary hyperphosphorylation of tau [2]. The comprehension of the role of Aβ in sporadic AD is more difficult as it deposits with age and injury [3]. Age-related amyloid accumulation presents a challenge to the diagnostic specificity of fluid or imaging biomarker evidence of amyloidosis beyond the 7th decade [4], which, in turn, casts uncertainty on its role in the pathogenesis of AD in these age ranges. Investigations using monoclonal antibodies to the fibrillar form of Aβ have yielded negative results in improving cognition in global randomized controlled trials but did reduce brain amyloid [5]. To clarify the effect of age on Aβ deposition in sporadic AD, we sought to explore the degree of amyloid burden in patients with sporadic young onset AD (YOAD); that is, with AD starting before the age of 65 years, where there is low background of age-related deposition, to test the null hypothesis that Aβ has no part in the molecular processes which result in AD. We also studied whether there were differences between men and women with respect to amyloid deposition and cognitive change in YOAD as there is evidence that women have more AD than men, possibly because women live longer and have different immune systems due to their greater propensity to autoimmune diseases [6]. In Australia, amyloid positron emission tomography (PET) scanning is not used to define AD and the ATN classification system is only used in clinical trials. Amyloid PET scanning is performed in young patients with the question of dementia where there is uncertainty as to the diagnosis.

## MATERIALS AND METHODS

In 2000, a 20-year investigation into the neurobiology of young onset dementia (YOD) was initiated, the ARTEMIS Project [7]. General practitioners, neurologists, psychiatrists, geriatricians, and other physicians referred patients with suspected YOD. YOD is defined as patients with dementia with onset prior to the age of 65 years. Patients, their carers, and their families completed 6-month reviews for a median of 10 years (3–15 years). The same neurological team diagnosed and managed the patients.

All patients and their carers gave written informed consent. Ethics approval for this research was received from Joondalup Health Campus Human Research Ethics Committee: JHC HREC: ARTEMIS 1406. All methods were performed in accordance with the relevant guidelines and regulations of the JHC HREC.

Patients were diagnosed on cognitive and behavioral symptoms associated with progressive functional decline. The NINCDS-ADRDA criteria was used for diagnosis [8]. Patients with psychiatric disease and/or delirium were excluded. The patient’s diagnosis and the criteria were reviewed at every visit. History of cognitive impairment was recorded from the patient and their informant. Cognitive assessments including: Addenbrooke’s Cognitive Examination (ACE)—both unrevised pre-2005 and revised 2005 assessments were used, Mini-Mental State Examination (MMSE), Total Functional Capacity, Symbol Digits Modalities Test, Depression Anxiety Stress Scale, and the Clock Drawing Test (CDT) were performed on the patients. The participants’ informants completed the Frontal Rating Scale and the Cambridge Behavioural Inventory assessments.

Neuropsychological tests were performed when diagnostic uncertainty existed. YOAD diagnosis refers to probable AD dementia [9]. The patient population only included individuals with functional decline, without mixed presentations to assure that co-existent cerebrovascular, Lewy body, or other neurological processes were not present. Presentations from medication side effects were also excluded.

Brain imaging modalities were supplementary to the diagnosis. Magnetic resonance imaging (MRI) and ^18^Fluorodeoxyglucose uptake were performed to improve the probability that AD pathophysiological process was present in the YOAD patients and to exclude other pathologies. Tau PET imaging was not possible at the time of the study. Biomarkers such as low cerebrospinal fluid Aβ_1–42_ were also not available.

The control groups consisted of healthy age and sex matched controls and patients with behavioral variant frontotemporal dementia (FTD) and Lewy body disease (LBD). At the time of compilation of the data for this study, we did not have patients with primary progressive aphasia, vascular dementia, prion disease, progressive supranuclear palsy, corticobasal degeneration, alcohol related dementia, or chronic traumatic encephalopathy to act as controls. Patients with the question of YOD were included, i.e., YOAD, FTD, and LBD. Patients with psychiatric disease or delirium were excluded. The criteria for diagnosis were developed over time [10–13]. The McKeith criteria was used for LBD [14]. YOAD patients had almost no deep white matter intensities: Fazekas score 0–1 [15].

Only patients with sporadic YOAD and FTD were used, excluding familial AD. PCR determined *APOE* genotyping [16]. Familial AD was excluded by the absence of family history and negative gene tests for the amyloid precursor protein gene and presenilins 1 & 2.

An Allegro GSP whole body PET camera was used. The standard dose of 370 MBq (10 mCi) ^11^C-Pittsburgh compound B (^11^C -PiB) 2-(4^′^-[^11^C] methylaminophenyl)-6-hydroxybenzothiazole (University of Pittsburgh/Uppsala University) was injected 40 min before performing the emission scans. No preparation or testing was required for the patient prior to injection. The camera clock and dose calibrator were synchronized for precise timing. For accurate acquisition of scans, movement of patients during and between emission and transmission of scans was avoided. Preliminary emission-only scout view was performed to verify correct patient position. A 6-min transmission scan followed. The emission scan results were obtained in list mode and reconstructed into summed static and dynamic 6×5-min series [17]. Computational analysis of PET by AIBL (CapAIBL) program (CSIRO) was used to perform semi-quantitative analysis. The differences in global standardized uptake value ratios (gSUVR) were made with reference to the cerebellum. PiB retention data in different anatomical regions were expressed as the PiB SUV region of interest divided by the SUVR for reference region. The difference in gSUVR, MMSE, ACE-R, and CDT by disease group was examined with non-parametric analysis (Wilcoxon/Mann-Whitney test) due to small numbers (*n* < 30 in both groups), only those p values corrected for multiple comparisons are presented. Comparisons in PiB retention in different anatomical regions between male, female, and control populations were analyzed using the Kruskal-Wallis test. The difference in *APOE* genotyping by disease group was examined by Fisher’s exact test.

## RESULTS

Table 1 shows age distribution for the YOAD group at onset, time of PET scan, and cognitive tests. Table 2 shows the amyloid binding and other data for men and women. There were 22 women with a mean gSUVR was 2.68±0.73. The mean MMSE, ACE–R, and CDT scores are shown, along with the number of patients who had at least one *APOE* ɛ4 allele (72.7%). Table 2 reveals similar data for 20 males with YOAD with a mean gSUVR of 2.46±0.55. The cognitive assessments for men are revealed in Table 2. 80% of men had at least one *APOE* ɛ4 allele. Men and women with YOAD had a significantly higher amyloid burden than disease and healthy controls (Table 2). There were no significant differences between men and women in amyloid uptake, cognitive assessments, nor APOE alleles (Table 3) (*p* = 0.26–0.74). There were no significant differences in PiB retention in different brain regions (Table 4). There was no significant difference between men and women in the frequency of *APOE* ɛ4 alleles (*p* = 0.72). The frequency of APOE ɛ4 alleles for men and women was greater than the control group (Table 2).

**Table 1:** Age of onset, positron emission tomography (PET), or cognitive tests among young onset Alzheimer’s disease patients by gender

	Total	Male	Female
N	42	20	22
	Mean (std)/Median	Mean (std)/Median	Mean (std)/Median
Age at onset	55.2 (4.03)/55.0	56.1 (3.67)/55.0	54.5 (4.26)/55.0
Age at PET	59.6 (5.36)/59.0	60.9 (4.53)/59.5	58.6 (5.91)/58.0
Age at cognitive test	59.3 (5.39)/59.0	60.4 (4.46)/59.5	58. 3(6.05)/57.5

**Table 2:** Differences in amyloid binding, cognitive data,

		YOAD disease			Wilcoxon^1^	Disease control population			Wilcoxon^1^	Healthy control population			Wilcoxon^1^
		*n*	Mean (Std)	Median	or Fisher’s	*n*	Mean (Std)	Median	or Fisher’s	*n*	Mean (Std)	Median	or Fisher
					exact test p				exact test p				exact test p
Female	gSUVR^2^	22	2.68 (0.73)	2.50	<0.0001	4	1.21 (0.3)	1.15	<0.0001	5	1.1 (0.3)	1.15	<0.0001
	MMSE^3^	22	18.18 (5.87)	18.0		4	20.4 (6.5)	24.0	0.12	5	27.3 (0.2)	26.3	<0.001
	ACE-R^4^	20	53.20 (19.87)	54.0		4	60.9 (25)	70.0	0.14	5	90.2 (5.0)	88.1	<0.001
	CDT^5^	21	1.48 (1.61)	1.0		4	2.5 (2.0)	2.2	0.12	5	4.5 (0.5)	4.2	<0.001
	APOE4+^2^	16	72.7%			4	22.0%		0.02	5	24.0%
Male	gSUVR^2^	20	2.46 (0.55)	2.50	<0.0002	4	1.30 (0.2)	1.20	<0.0002	5	1.2 (0.2)	1.23	<0.0002
	MMSE^3^	20	18.55 (7.39)	19.0		4	21.3 (3.2)	23.0	0.15	5	29.2 (0.1)	28.1	<0.001
	ACE-R^4^	19	59.89 (21.0)	60.0		4	64.2 (20)	65.2	0.11	5	89.2 (2.0)	87.5	<0.001
	CDT^5^	21	1.74 (1.67)	1.0		4	2.7 (2.1)	2.4	0.21	5	4.3 (0.2)	4.1	<0.001
	APOE4+^2^	16	80.0%			4	26.0%		0.15	5	23.2%

^1^Wilcoxon test (Mann-Whitney test) for non-parametric two sample *t* test. ^2^ApoE4+ difference between groups were performed by Fisher’s Exact test. gSUVR, Global standardized uptake value ratios; MMSE, Mini-Mental State Examination; ACE-R, Addenbrooke’s cognitive examination-revised; CDT, Clock drawing test.

**Table 3:** Amyloid binding, cognitive data, and APOE genotyping for males and females with YOAD

	Male (AD)	Female (AD)					Wilcoxon^1^ or
	*n*	Mean (Std)	Median	*n*	Mean (Std)	Median	Fisher exact test *p*
gSUVR	20	2.37 (0.54)	2.50	22	2.68 (0.73)	0.73	0.26
MMSE	20	18.55 (7.39)	19	22	18.18 (5.87)	5.87	0.74
ACE-R	19	59.89 (21.61)	60	20	53.20 (19.87)	19.87	0.27
CDT	19	1.74 (1.67)	1	21	1.48 (1.61)	1.61	0.57
ApoE4+^2^	16 (80%)			16 (72.7%)			0.72

^1^Wilcoxon test (Mann-Whitney test) for non-parametric two sample *t* test. ^2^ApoE4+ difference between groups were performed by Fisher’s Exact test. gSUVR, Global standardized uptake value ratios; MMSE, Mini-Mental State Examination; ACE-R, Addenbrooke’s cognitive examination-revised; CDT, Clock drawing test.

**Table 4:** Comparison of PiB retention in different brain regions^+*^

	Frontal cortex	Parietal cortex	Temporal cortex	Occipital cortex	Striatum	Cerebellum
YOAD –males (*n* = 20)	2.5±0.3	2.5±0.2	2.0±0.3	1.9±0.2	2.4±0.3	1.1±0.1
YOAD –females (*n* = 22)	2.3±0.4	2.4±0.3	1.9±0.2	2.2±0.3	2.3±0.1	1.05±0.05
Healthy controls –males (*n* = 5)	1.2±0.2	1.3±0.1	1.1±0.2	1.2±0.2	1.3±0.1	1.15±0.1
Healthy controls –females (*n* = 5)	1.3±0.1	1.4±0.2	1.2±0.1	1.4±0.3	1.2±0.2	1.12±0.2
Disease controls –males (*n* = 4)	1.3±0.1	1.1±0.2	1.3±0.1	1.3±0.3	1.2±0.2	1.2±0.1
Disease controls –females (*n* = 4)	1.1±0.2	1.2±0.3	1.4±0.2	1.1±0.1	1.3±0.1	1.1±0.2

^+^PiB SUV region of interest/reference region: SUV, standard uptake values; reference region, cerebellum. ^*^Kruskal-Wallis test *p* > 0.2–0.4 for all comparisons between male, female with YOAD; and *p* = 0.001–0.05 for all regions male, female compared to healthy and disease controls.

There were no significant cognitive testing differences between both sexes with YOAD and the disease controls, but there were between YOAD and the healthy controls (Table 2). The amyloid binding for men and women with YOAD, when combined, is significantly greater than the controls (*p* = 0.0003).

## DISCUSSION

We have found that amyloid uptake in the brains of people with YOAD is increased in comparison with the control groups. There were no differences between men and women with YOAD, both revealing elevated amyloid binding in contrast to controls. There was no difference in the amount of the amyloid binding between men and women and no dissimilarity in the extent of cognitive dysfunction. Both sexes showed a high prevalence of an *APOE* ɛ4 allele, without a major difference between men and women.

In our previous studies we have found that YOAD has differences between old onset disease in relation to cerebrovascular risk factors and ethnicity providing support for the notion that YOAD may have a divergent pathophysiological origin [18]. Our results show that YOAD shares an augmented amyloid binding similar to old onset disease and this, in some way, correlates with the presence of *APOE* ɛ4 alleles. Other investigations have conveyed that the amyloid deposits in the brains of aging people such that by the age of 70 years 40–50% of cognitively normal people have amyloid accumulation, which increases with each decade [19], limiting amyloid deposits diagnostic usefulness pathologically and in amyloid PET imaging with age [20]. Additional pathologies, such as stroke and head injury, complicate the picture of old onset disease, to which the amyloid deposition may be a reaction and not central to the AD pathophysiological pathway [21]. The study of YOAD negates these potentially conflicting variables and supports the hypotheses that amyloid deposition is fundamental in the pathophysiology of AD in young adults and that *APOE* ɛ4 is involved [22]. Furthermore, the combination of increased amyloid binding and the presence of *APOE* ɛ4 alleles being a useful diagnostic laboratory confirmation of the diseases. The novelty and added value of our study, in comparison to previous studies, is the emphasis on YOAD only in patients without evidence of vascular or other pathologies.

Whether YOAD is truly divergent from late onset AD requires further investigation as some studies suggest differences [23], but others suggest that YOAD is on a continuum with old onset and that the distinction may be artificial [24]. We have observed differences in cerebrovascular risk factors and ethnicity [18, 25]. Others support a spectrum of YOAD and that emphasis of genetic variants such as the prion protein gene (PRNP) and the microtubular associated protein tau (MAPT) in AD plus studies of biofluids, multimodal imaging, and other methodologies may help to gain insight into YOAD [26, 27]. Furthermore, the investigation of the amyloid plaque proteome in YOAD and Down syndrome using laser capture microdissection suggest that lysosomal proteins, like secreted modular calcium-binding protein 1, phosphorylated Aβ, and pyroglutamate Aβ may be important divergents to neuritic plaques from old onset patients, suggesting therapeutic possibilities [28].

Amyloid deposition along with the hyperphosphorylation of the microtubular associated protein tau and neuronal loss represent the pathological hallmarks of AD and these three variables contribute to the dementia syndrome [29]. Approximately 30% of cognitively normal elderly people have AD at neuropathological examination [30]. Amyloid deposition is common among the elderly—especially in the seventh and eighth decades and beyond [31, 32]. The studies of Jack et al. indicate that normal people under the age of 60 years have a low probability of amyloid deposition using PET techniques and a low frequency of *APOE* ɛ4 alleles, approximately 20% [33]. The SUVR in young adults is usually below the cut-off 1.42. These observations support our findings that young demented adults with AD have prominent amyloid deposition raising the clinical probability that amyloid deposition and its detection by PET imaging techniques *in vivo* represent part of the definitive pathophysiological pathway of AD, and not an epiphenomenon of aging. Our discovery redefines amyloid as center-stage of the pathological and chemical reactions resulting in AD and affirms the amyloid hypothesis [34]. This hypothesis has come in for recent criticism in that several trials of monoclonal antibodies—Solanezumab, Crenezumab, Aducanumab, and others—to the Aβ protein have provided negative results in global randomized controlled clinical trials [35]. However, a recent reanalysis of the Aducanumab study (the phase III EMERGE trial) looked at an additional three months of data with patients on high-dose Aducanumab and observed statistically significant changes in the Clinical Dementia Rating-Sum of Boxes score with *p* = 0.010–0.031, with the high dose group achieving secondary endpoints, refuting the earlier futility analysis [36]. The patients in the re-analysis *APOE* ɛ4 stratification allowed higher doses and longer treatment, explaining the findings. Lecanemab, a monoclonal antibody to Aβ soluble protofibrils, has been shown to reduce amyloid and result in less cognitive decline in a randomized control trial (Clarity AD Clinical Trial), strengthening our findings [37].

Our data, which reveals that YOAD patients had greater amyloid burden and *APOE* ɛ4 alleles than controls, provides strong evidence that *APOE* ɛ4 is a driving force for amyloid deposition in YOAD, and is consistent with the observations that *APOE* ɛ4 lowers the age of onset of AD especially if there is a family history [38]. It is possible that heterogeneity in clinical trial data in AD may be because under the age of 65 years AD is driven by amyloid, whereas over 65 years amyloid may be part of protective neurochemical reaction to AD pathology, tau and other factors may be more operational.

Our observations support those of Zwan et al., that amyloid PET imaging is useful in the diagnosis of YOD, increasing diagnostic certainty and aiding management [39]. The meta-analysis performed by Ossenkoppele et al. indicated that, like our data, amyloid PET positivity related to diagnosis, age, and *APOE* genotype, and may be especially useful in those who are *APOE* ɛ4 negative and have an early onset [40]. That study underscored the likelihood of detecting incidental amyloid pathology with advancing age, limiting its usefulness and that there is low amyloid burden in non-Alzheimer pathologies like LBD and FTD. Other investigations have highlighted the value of amyloid PET imaging in YOAD and unusual dementias especially when cerebrospinal fluid Aβ and tau are equivocal [41]. These observations provide further weight to the diagnostic work-up of YOD as provided by others [42]. Hellwig and colleagues confirm the value of amyloid PET in increasing diagnostic accuracy in AD [43]. Our results suggest that this is especially so in young adults.

The major limitations of this study are the relatively small N numbers in each patient group. The ABIDE project provided additional evidence that amyloid positive and negative scans were associated with useful changes in diagnosis and treatment in those with and without dementia [44]. In the future, tau PET imaging may be a useful adjunct to amyloid imaging in diagnosing and tracking YOAD, especially those at genetic risk [45]. The investigations of Benzinger et al. suggest there are regional differences in the brain in amyloid deposition, hypometabolism, and atrophy and that not all regions of atrophy show reduced metabolism even in the presence of amyloid deposition [46], illustrating the difficulty in comprehending the pathophysiology of AD, and underpinning the importance of amyloid deposition in the pathophysiology of sporadic YOAD which, in the future, will be aided by the application of tau and microglial activation markers using PET imaging in longitudinal studies [47].

### Conclusion

In conclusion, our survey suggests that in sporadic YOAD amyloid deposition and *APOE* ɛ4 interact and are fundamental in its pathogenesis. Our findings raise the possibility that young onset AD may be driven more by Aβ than older onset disease, which might explain the heterogeneity in clinical trial data using anti-amyloid treatments.

## Data Availability

All data generated or analyzed during this study are included in this published article. More information on the study protocol and raw data is available on request to the corresponding author.
